# STAKEHOLDER PERSPECTIVES ON QUALITY DEMENTIA CARE IN LOW-RESOURCE LONG-TERM CARE SETTINGS

**DOI:** 10.1093/geroni/igad104.1900

**Published:** 2023-12-21

**Authors:** Alison Rataj, Sarah Holmes, Nancy Kusmaul, Laura Davie, Yoon Kim, Michael Lepore

**Affiliations:** University of New Hampshire, Durham, New Hampshire, United States; University of Maryland School of Nursing, Baltimore, Maryland, United States; University of Maryland Baltimore County, Baltimore, Maryland, United States; University of New Hampshire, Durham, New Hampshire, United States; University of Maryland Baltimore, Baltimore, Maryland, United States; University of Maryland School of Nursing, Baltimore, Maryland, United States

## Abstract

Having dementia care staff who are knowledgeable about the needs and preferences of residents living with dementia (RLWD) in long-term care (LTC) settings holds promise for supporting quality of care. However, staff who work in low-resource LTC settings tend to have fewer opportunities to get to know the residents, who in turn experience increased risk of adverse outcomes. Low-resource LTC settings can experience barriers in providing quality dementia care due to their limited ability to capture pertinent information about residents’ needs and preferences and ensuring that information is known by dementia care staff. We sought to describe stakeholder perspectives regarding the collection and sharing of information about RLWD in four low-resource LTC settings (two nursing home; two assisted living) that serve RLWD. All four settings were in medically underserved areas with two rural settings in New Hampshire and two urban settings in Maryland. Preliminary site visits conducted revealed strategies used by LTC providers to recruit and retain dementia care staff. In-depth semi-structured interviews were conducted with a purposive sample of stakeholders (administrative leaders, direct care staff, RLWD and family members). Interviews were transcribed and thematically analyzed in NVivo12. Themes were identified in four core topic areas: 1) identifying information about RLWD to support quality care; 2) finding and accessing information by the care team; 3) sharing information with RLWD; and 4) describing quality measures most relevant for RLWD. Findings shed light on practical strategies used by low-resource LTC settings and motivate future research on measuring dementia care quality.

